# Preliminary findings on associations between moral emotions and social behavior in young children with normal hearing and with cochlear implants

**DOI:** 10.1007/s00787-015-0688-2

**Published:** 2015-02-21

**Authors:** Lizet Ketelaar, Carin H. Wiefferink, Johan H. M. Frijns, Evelien Broekhof, Carolien Rieffe

**Affiliations:** 1 Department of Developmental Psychology, Leiden University, PO Box 9555, 2300 RB Leiden, The Netherlands; 2 Dutch Foundation for the Deaf and Hard of Hearing Child, Amsterdam, The Netherlands; 3 Department of Otorhinolaryngology and Head and Neck Surgery, Leiden University Medical Center, Leiden, The Netherlands; 4 Leiden Institute for Brain and Cognition, Leiden University, Leiden, The Netherlands

**Keywords:** Moral emotions, Deafness, Cochlear implant, Behavior problems, Social competence

## Abstract

Moral emotions such as shame, guilt and pride are the result of an evaluation of the own behavior as (morally) right or wrong. The capacity to experience moral emotions is thought to be an important driving force behind socially appropriate behavior. The relationship between moral emotions and social behavior in young children has not been studied extensively in normally hearing (NH) children, let alone in those with a hearing impairment. This study compared young children with hearing impairments who have a cochlear implant (CI) to NH peers regarding the extent to which they display moral emotions, and how this relates to their social functioning and language skills. Responses of 184 NH children and 60 children with CI (14–61 months old) to shame-/guilt- and pride-inducing events were observed. Parents reported on their children’s social competence and externalizing behavior, and experimenters observed children’s cooperative behavior. To examine the role of communication in the development of moral emotions and social behavior, children’s language skills were assessed. Results show that children with CI displayed moral emotions to a lesser degree than NH children. An association between moral emotions and social functioning was found in the NH group, but not in the CI group. General language skills were unrelated to moral emotions in the CI group, yet emotion vocabulary was related to social functioning in both groups of children. We conclude that facilitating emotion language skills has the potential to promote children’s social functioning, and could contribute to a decrease in behavioral problems in children with CI specifically. Future studies should examine in greater detail which factors are associated with the development of moral emotions, particularly in children with CI. Some possible directions for future research are discussed.

## Introduction

Research in the normally hearing (NH) population has demonstrated that moral emotions such as shame, guilt, and pride are important determinants of social competence, reflected in being liked by others, for example [1, 2]. Conversely, an impaired moral development is associated with a range of undesirable behaviors in preadolescents, including bullying and aggression [3–5] and, at the far end of the spectrum, even criminal behavior and psychopathy in adolescents and adults [6, 7]. These studies indicate that moral emotions play a significant role in regulating social behavior, but whether this association can already be observed in early childhood is unclear. Although literature concerning the development of moral emotions in young children is abundant (e.g., [1, 8, 9]), few studies have examined the relationship between moral emotions and social behavior in early childhood. The general consensus among emotion theorists is that moral emotions start to develop in early toddlerhood but that children’s ability to experience and regulate these emotions increases in the next couple of years (cf. [8]). For example, Nunner-Winkler [10] found that 8-year-olds, but not 4-year-olds, expect negative feelings after a moral transgression. This seems to imply that although young children are able to experience a moral emotion, their ability to anticipate the consequences of their behavior is still limited. Consequently, moral emotions might not regulate young children’s social behavior to the same extent as observed in older children and adults.

The ability of children with hearing impairments to experience and express moral emotions, and how this ability relates to their social functioning, has received little or no attention to date even though social problems are known to exist in this population. Children and adolescents with prelingual, severe to profound hearing loss more often experience social difficulties than NH peers, which is manifested in problematic peer relations [11], behavior problems [12], and symptoms which may be precursors of behavior disorder and antisocial personality (i.e., poor impulse control, lack of empathy) [13]. These days, the vast majority of young children with severe to profound hearing loss born in Western countries receive a cochlear implant (CI), often before their second birthday [14, 15]. This electronic device bypasses the damaged part of the ear by directly stimulating the auditory nerve which, combined with extensive rehabilitation, enables sound perception and in turn could benefit spoken language skills [16, 17]. Yet, how cochlear implantation affects these young children’s social functioning and which factors underlie their social development is largely unknown. Determining risk or protective factors is important in light of optimizing rehabilitation programs for children with CI. This study is the first to explore the moral development of children with CI and to examine its relationship with these children’s social functioning.

## Function and development of moral emotions

Emotions have a social function, motivating people to find a balance between their own interests and certain social requirements to optimize interpersonal relations [18, 19]. A number of theorists have argued that moral emotions take a special position in the spectrum of emotions (e.g., [1, 20–24]). Crucially, moral emotions include a self-evaluative component, and occur when people judge their own behavior as (morally) right or wrong [8]. This self-awareness or self-reflection may motivate people to correct their own behavior or even better, to prevent themselves from committing moral transgressions in the future [1, 21]. Each moral emotion has its own function and a corresponding pattern of behavior. Although the same event may cause shame in one person and guilt in the next [21, 25], a distinction between these two emotions can be made based on appraisals concerning stability and globality [24, 25]. Shame arises when a failure or transgression is attributed to a global and stable cause (e.g., ‘I broke the vase because I am clumsy’). Guilt, on the other hand, is caused by specific and unstable attributions (e.g., ‘I broke the vase because I did not look where I was going’). Both emotions communicate to others that you are aware of your transgression and feel bad, yet shame is associated with escape-related behavior (e.g., making yourself smaller, avoiding eye contact), whereas guilt is associated with reparative behavior (e.g., apologizing, trying to undo the consequences) [1, 21, 25]. Pride can be felt when you have accomplished something notable, and makes you want to repeat or sustain the behavior that led up to this emotion. Its expression is aimed at drawing attention to this accomplishment (e.g., expanded posture, making eye contact), and signals to others that you (temporarily) deserve a higher status within the group [22, 26]. In sum, moral emotions discourage inappropriate (i.e., morally incorrect) and reinforce appropriate (i.e., morally correct) behavior. Therefore, it is not surprising that emotionally competent NH children—those who know when and how to express (moral) emotions—are generally also perceived to be more socially competent [1, 2, 27].

Moral emotions require certain insights and capacities, which develop over time. Children need to be aware of the dominant moral standards and have to be able to evaluate their own behavior in this context [8, 21, 23–25]. Having a sense of self is a prerequisite for the ability to reflect upon one’s own behavior. Self-awareness typically develops during the second year of life, which is illustrated by self-referential behaviors such as the use of personal pronouns (i.e., I, me, myself, mine) and self-recognition [8, 25]. Parents play an important role in children’s developing sense of self. By providing feedback on their children’s behavior, parents direct children’s attention to their actions, helping them to reflect upon, evaluate, and ultimately regulate their behavior [8]. Yet, an overly critical attitude toward their children’s behavior could lead to shame proneness, which in turn could impact children’s development of self [cf. 9]. During the toddler period, most NH children become increasingly able to evaluate their own behavior based on what they have learned from previous feedback and will start to generalize this knowledge to other situations. From 3 years onwards, NH children start to internalize a personal set of moral standards that will eventually channel their (emotional) behavior, independent from outside guidance [9, 23–25].

In order for a personal set of moral standards to develop, children need to be able to judge their own behavior through other people’s eyes, which requires certain socio-cognitive abilities. The best-known example of these is the so-called Theory of Mind (ToM), which entails the capacity to take other people’s perspective into account [28]. The majority of NH children show a major development in their ToM understanding between the ages of 2 and 5 years old [29], but ToM skills of children with CI are known to fall behind during this crucial period. In early and middle childhood, children with CI are typically less able than their NH peers to predict other people’s behavior based on these people’s desires and expectations, but tend to use their own frame of reference instead [30, 31]. A limited understanding of other people’s perspectives also implies a limited ability to anticipate other people’s judgments of their behavior. Consequently, children with CI may be less inclined to express moral emotions because they do not realize that they have done something that would be judged as reprehensible or admirable by others. Also in the context of overt feedback on their behavior, children with CI could still experience and express moral emotions to a lesser extent than NH children. Studies have shown that children with CI more often than their NH peers have difficulties recognizing other people’s emotions [32, 33] and are less sensitive to intonation [34]. Therefore, these children might not pick up on more subtle forms of feedback, which are relayed by someone’s facial expression or tone of voice, for example. If children with CI are indeed less aware of other people’s evaluations of their behavior, this could hamper the process of internalizing moral standards.

## The role of communication and socialization

Emotions are subjective experiences in response to meaningful events. Yet, how these emotions are interpreted and displayed is modulated by the social environment [1, 18]. For example, play with other children provides a platform for learning to regulate emotions and for practicing social skills. In addition, parents act as role models and instructors, providing examples of appropriate behavior and correcting children’s (emotional) behavior if necessary. Children need to pick up on cues in their environment in order to form a conception of how they should behave, which emotions to experience and when and how to express these emotions. These cues are communicated in various ways, such as through body language, eye contact, facial expressions, language content, and tone of voice. For example, a parent may provide verbal feedback to correct a child’s behavior (“do not hit your brother”) or may show a disapproving facial expression. Both acts of communication convey the same message. On the other hand, the same sentence can carry different meanings dependent upon tone of voice.

Children with CI are less likely to pick up on these cues from the environment. Even though a CI enables sound perception, a large proportion of these children still faces language delays [16], which is problematic given that at least 90 % of deaf children are born into hearing families [35]. Parents of children with hearing impairments more often have difficulties conversing with their children, particularly about abstract topics [36]. This includes having conversations about emotions; helping children to label their emotions and discussing how to communicate their emotions appropriately. Having a limited ability to identify own and other people’s emotions is likely to also hinder children’s ability to interpret nonverbal messages. For example, facial expressions may not have much meaning for children if they have not learned how to interpret these. Moreover, limited emotion knowledge also leads to difficulties in verbally communicating emotions during interactions with others. This in turn could lead to misinterpretation of the intended message by the other party, potentially damaging the relationship [27].

In addition to communication difficulties that can arise in situations where children with CI interact with other people, these communication difficulties will also play a role when the child with CI is not directly addressed. In other words, children with CI will also more often miss out on opportunities for so-called incidental learning, i.e., overhearing conversations between others, as has been shown for children with hearing impairments without a CI [37]. Particularly in noisy situations, for example in playgrounds or at family gatherings, children with hearing impairments are known to benefit less from their CI [38]. And even when children with CI are addressed directly, they will often have to rely on visual as well as auditory cues, which means they need to face the person who is talking to them. This makes it hard for them to simultaneously focus on the object or event this person is talking about (cf. [39]). An impoverished quality of interactions with NH people in their immediate surroundings combined with limited opportunities for incidental learning could negatively impact these children’s ability to develop moral emotions.

## Current study

This study’s first aim is to examine the extent to which young children with NH or with CI display moral emotions in an experimental setting. Because of communication difficulties and limited opportunities for incidental learning [36, 37], children with CI presumably have had less opportunity to learn and internalize moral standards, and subsequent moral emotions. Moreover, compared with NH children, children with CI more often have an impaired insight into other people’s emotions and perspective [30–33], which further hampers their ability to make inferences about their own behavior from cues in their environment. Therefore, we expect children with CI to display moral emotions to a lesser degree than NH peers.

Secondly, the associations between moral emotions and social behavior are examined. Because moral emotions play such a crucial role in social functioning of the NH population at later ages [2, 3, 7], we could expect to observe this relationship already early in life, both in the NH group and in the CI group. Alternatively, it could be that young children (regardless of hearing status) cannot yet anticipate moral emotions to follow from their behavior [10], which would imply that in both groups of children in this study moral emotions are not related to social behavior.

Thirdly, we wish to verify whether communication indeed plays an important role in the development of moral emotions. We examine children’s language skills as a determinant of communication. Particularly emotion-related language might be important for children’s social–emotional development. Regardless of children’s hearing status, we expect to find a positive relationship between their ability to understand and use emotion language and the extent to which they express moral emotions. Within the CI group, we also assess whether general spoken language abilities are related to moral emotions.

Fourthly, we explore whether earlier implantation promotes children’s social and emotional functioning similar to what has been found for their spoken language skills [16].

## Methods

### Participants

This study is part of a larger research project, which focuses on various areas of the social–emotional development of young children with CI and with NH. A total of 244 children (60 with CI and 184 with NH) from the Netherlands and the Dutch-speaking part of Belgium participated in this study. All children were born to hearing parents and had no apparent mental health disorders such as ADHD or autism spectrum disorders. Characteristics of the samples are reported in Table 1. Age, gender, and socioeconomic status (based on maternal education and net household income) did not differ between the groups. All children with CI had prelingual, severe to profound hearing loss and had received their (first) implant before the age of 3 years. Sixty-eight percent had been using their CI for 12 months or more at the time of data collection. Parents indicated that practically all children were wearing their CI full-time. Approximately half the group had two implants and the other half had one. All children with CI entered a tailored rehabilitation program after implantation, which includes joining specialized playgroups, receiving technical support for the device, speech therapy, and visits to a psychologist. At the time of data collection, 22 children (37 %) preferred solely to use spoken language, the remaining 38 children (63 %) preferred to use some form of signed language, mostly sign-supported Dutch (i.e., spoken Dutch supported by signs).

**Table 1:** Sample characteristics

	CI (*n* = 60)	NH (*n* = 184)
Age, mean (SD), months	38 (14.3)	38 (12.1)
Age, range, months	14–61	14–61
Male, no. (%)	36 (60 %)	110 (60 %)
Socioeconomic status		
Maternal education, mean (SD)^a^	3.46 (0.83)	3.63 (0.62)
Net household income, mean (SD)^b^	3.68 (1.12)	3.65 (0.96)
Age at implantation, mean (SD), months	16 (7.3)	
Age at implantation, range, months	6–35	
Time with (first) CI, mean (SD), months	21 (12.8)	
Time with (first) CI, range, months	1–44	

^a^1 = no/primary education, 2 = lower general secondary education, 3 = higher general secondary education, 4 = college/university

^b^1 = <€15,000, 2 = €15,000–€30,000, 3 = €30,000–€45,000, 4 = €45,000–€60,000, 5 = >€60,000

## Materials

### Indices for moral emotions

#### Shame/guilt

Three tasks were designed to evoke feelings of shame/guilt. In the Broken Car Task, children were led to believe they had broken the experimenter’s toy car (i.e., the wheels would come off when children played with it). The other two tasks involved failure on an assignment that appeared to be easy, which supposedly evokes shame/guilt [23]. In the Copy Task, children were asked to copy a drawing made by the experimenter. Children always received negative feedback upon completion of their drawing. In the Bottle Task, the experimenter asked children to open a bottle that, unknowingly to the child, was equipped with a childproof safety cap. The experimenter opened and closed the bottle before handing it to the child, demonstrating it could be opened easily. Based on previous research [1, 23, 40], the occurrence of the following four behaviors was coded on a three-point scale (0 = not at all, 1 = a little, 2 = a lot) for each of the tasks: (1) negative response to the situation, (2) gaze aversion/turning away from situation, (3) collapsed body, and (4) corners of the mouth turned down/lower lip pushed outward (pouting). Hiding one’s face was also scored but showed a floor effect for all tasks and was removed from the scale. A single, overall score (ranging between 0 and 2) for shame/guilt was computed by averaging the ratings of the four items across the three tasks. The reliability of the scale meets the expected minimum of Cronbach’s alpha >0.70 [41] (Table 2).

**Table 2:** Internal consistencies, means, and SDs for measures of social and emotional functioning

	No. of items	Min–Max	Cronbach’s alpha	Inter-item correlation	CI (*n* = 60)	NH (*n* = 184)
					*M* (SD)	*M* (SD)
Moral emotions						
Shame/guilt***	12	0–2	0.79	0.24	0.19 (0.19)	0.41 (0.33)
Pride*	6	0–2	0.81	0.41	0.70 (0.49)	0.89 (0.54)
Language						
Emotion vocabulary***	20	0–1	0.92	0.37	0.46 (0.26)	0.57 (0.28)
Language understanding					86.49 (17.59)	
Word production					89.08 (18.67)	
Sentence production					84.09 (14.47)	
Social functioning						
Social competence	10	0–2	0.70	0.17	1.42 (0.35)	1.48 (0.33)
Externalizing behavior	10	0–2	0.71	0.20	0.61 (0.38)	0.53 (0.31)
Cooperation	9	0–2	0.87	0.43	1.62 (0.41)	1.62 (0.43)

* *p* < 0.05, ** *p* < 0.01, *** *p* < 0.001

#### Pride

Two pride-evoking tasks were administered, both of which involved mastery. These two tasks directly followed their shame-/guilt-evoking counterparts, which would have set the stage for children to believe that these tasks were hard. Mastering them this time was assumed to evoke pride [23]. In the Copy Task, children again were asked to copy a drawing but this time were given positive feedback. In the Bottle Task, the experimenter looked at the bottle that the child had been unable to open and ‘discovered’ that the cap was screwed on wrong. She then (without the child noticing) released the safety lock before handing the bottle back and encouraging the child to try again. The experimenter made sure children succeeded to open the bottle this time. Based on previous studies [23, 26, 40], three separate cues for pride were scored on a three-point scale (0 = not at all, 1 = a little, 2 = a lot): (1) positive response to situation, (2) smiling/laughing, and (3) eye contact and erect posture. Pointing to the outcome or applauding was also scored but showed a floor effect for both pride tasks and was removed from the scales. A single, overall score (ranging between 0 and 2) for pride was computed by averaging the ratings of the three items across the two tasks. The reliability of the scale meets the expected minimum of Cronbach’s alpha >0.70 [41] (Table 2).

### Indices for social functioning

#### Social competence

Social competence was assessed by calculating mean scores for the items of the Prosocial and Peer Problems scales (5 items each) from the Dutch parent-report version of the Strengths and Difficulties Questionnaire (SDQ) [42, 43]. Parents rated each item on a three-point scale (0 = not true, 1 = somewhat true, 2 = certainly true). Negatively formulated items on the Peer Problems scale were reversed so that higher scores were indicative of less peer problems. The reliability of the scale meets the expected minimum of Cronbach’s alpha >0.70 [41] (Table 2).

#### Cooperation

Following each test session, experimenters completed a questionnaire that was designed for the purpose of this study concerning the child’s behavior during the test session. The scale Cooperation (9 items) reflects the extent to which children were motivated to complete the tasks and how responsive they were to the experimenter’s instructions. Items were rated on a three-point scale (0 = not, 1 = sometimes, 2 = often), mean scores across the items were calculated. The reliability of the scale meets the expected minimum of Cronbach’s alpha >0.70 [41] (Table 2). Data were missing for one NH child.

#### Externalizing behavior

Externalizing behavior was assessed by calculating mean scores for the Hyperactivity and Behavioral Problems scales (5 items each) from the Dutch parent-report version of the SDQ [42, 43]. Parents rated each item on a three-point scale (0 = not true, 1 = somewhat true, 2 = certainly true). The reliability of the scale meets the expected minimum of Cronbach’s alpha >0.70 [41] (Table 2).

### Indices for language

#### Emotion vocabulary

Children’s emotion language was measured with the Emotion Vocabulary Questionnaire, a parent-report measure that was designed for the purpose of this study. Parents rated whether their children knew and used (either in spoken or sign language) each of 20 emotion and/or mental state words (0 = no, 1 = yes). Basic emotions such as happy or angry, more complex emotions such as jealous or disappointed, and mental states such as dreaming or thinking were represented in the questionnaire. A mean score across the items was calculated to indicate children’s emotion vocabulary. The reliability of the scale meets the expected minimum of Cronbach’s alpha >0.70 [41] (Table 2). Data were missing for one child with CI.

#### Spoken language understanding and production

Spoken language understanding and production scores of children with CI were obtained via records from hospitals and counseling services. Part of the rehabilitation process after implantation involves monitoring children’s language development, most commonly by administering the Dutch versions of the Reynell Developmental Language Scales for language understanding and the Schlichting Expressive Language Test for word and sentence production [44]. Hospitals and counseling services were asked to provide children’s most recent scores. Recent language scores were unavailable for 11 children, and the language skills of 5 additional children were assessed with alternative, incomparable instruments, leaving us with language scores of 43 children with CI (Table 2). Using independent sample *t* tests, we compared children in the CI group with and without language scores and found no differences regarding age or any of the indices for social–emotional functioning.

## Procedure

Children with NH were recruited through day-care centers, preschools, and elementary schools in the Netherlands. Children with CI were recruited through hospitals and family counseling services all over the Netherlands and the Dutch-speaking part of Belgium. All children were tested individually in a quiet room at home, school or hospital. The emotion-evoking events were interspersed among other tasks not presented in this manuscript. Parents filled in questionnaires. Additional information, such as household income and age at implantation, was obtained from parents and/or medical records. Informed consent was obtained for all children and the study was approved by the University’s Medical Ethics Committee.

The tasks were nonverbal in nature (common gestures made clear what was expected) and did not require a verbal response from the children. The tasks were administered to children in the CI group by one of two hearing experimenters who were fluent in Dutch sign language and sign-supported Dutch. Children with CI were addressed in their preferred mode of communication (spoken or signed language) during the test session.

## Results

### Group differences on moral emotions and social functioning

To examine group differences for moral emotion expressions, social functioning and language, ANCOVAs controlling for age were performed. The ANCOVA for shame/guilt revealed a main effect for group (NH, CI), *F*(1, 241) = 27.38, *p* < .001, Cohen’s *d* = 0.84. Similarly, the ANCOVA for pride revealed a main effect for group (NH, CI), *F*(1, 241) = 6.47, *p* = .012, Cohen’s *d* = 0.39. Mean scores per group (Table 2) indicate that children with CI expressed moral emotions to a lesser extent than NH children. No main effects for group (NH, CI) were found for any of the measures of social functioning (Social competence: *F*(1, 241) = 1.70, *p* = .194, Cohen’s *d* = 0.18; cooperation: *F*(1, 240) = 0.00, *p* = .990, Cohen’s *d* = 0.00; externalizing behavior: *F*(1, 241) = 3.11, *p* = .079, Cohen’s *d* = 0.23). In addition, the ANCOVA for emotion vocabulary showed a main effect for group (NH, CI), *F*(1, 240) = 16.48, *p* < .001, Cohen’s *d* = 0.41, indicating that NH children knew and used more emotion words than children with CI. Spoken language skills were only obtained for children with CI. On average, children with CI scored 1 SD below the normative mean (*M* = 100, SD = 15) (Table 2).

### Associations between moral emotions and social functioning, and the role of language

Shame/guilt and pride increased with age in both groups of children. Cooperation also increased with age in both groups, whereas social competence only increased with age in the NH group. Externalizing behavior was unrelated to age in the NH group, but increased with age in the CI group (Table 3). Because age was a confounding variable, correlations between moral emotions and social functioning were calculated corrected for age (Table 4). Results for the NH group show that shame/guilt was related to social competence and that pride was related to both social competence and cooperation. Moral emotions were unrelated to externalizing behavior in the NH group. Results for the CI group show that moral emotions were unrelated to social functioning.

**Table 3:** Pearson correlations of moral emotions, social functioning, and language with age and implantation timing

	Chronological age		Age at implantation^a^	Time with CI^a^
	NH	CI	CI	CI
Moral emotions				
Shame/guilt	0.33***	0.40**	0.00	−0.01
Pride	0.25**	0.40**	−0.33*	0.35**
Social functioning				
Social competence	0.34***	0.09	−0.18	0.17
Cooperation	0.51***	0.55***	−0.18	0.17
Externalizing behavior	−0.09	0.34**	0.21	−0.21
Language				
Emotion vocabulary	0.76***	0.73***	−0.26	0.25
Language understanding		0.17	−0.48**	0.47**
Word production		0.14	−0.50**	0.48**
Sentence production		0.04	−0.58***	0.57***

* *p* < 0.05, ** *p* < 0.01, *** *p* < 0.001 (two tailed)

^a^Corrected for chronological age

**Table 4:** Pearson correlations of moral emotions with social functioning per group, corrected for age

	Shame/guilt	Pride	Social competence	Cooperation	Externalizing behavior
Shame/guilt	–	0.19	0.10	0.23	−0.10
Pride	0.27***	–	−0.04	0.12	0.06
Social competence	0.29***	0.18*	–	0.12	−0.41***
Cooperation	0.03	0.16*	0.19*	–	−0.12
Externalizing behavior	−0.10	0.00	−0.41***	−0.18	–

Right upper corner shows correlations for CI group, left lower corner shows correlations for NH group

* *p* < 0.05, ** *p* < 0.01, *** *p* < 0.001 (two tailed)

Next, we explored the association between various indices of language and the expression of moral emotions, as well as social functioning (Table 5). Emotion language, as indexed by emotion vocabulary, was unrelated to moral emotions in both groups. Yet, emotion vocabulary appeared to be positively related to social competence in both groups, and negatively related to externalizing behavior in the CI group. None of the indices for spoken language skills in the CI group were related to moral emotions or social functioning.

**Table 5:** Pearson correlations of language indices with moral emotions and social functioning, corrected for age

	Shame/guilt	Pride	Social competence	Cooperation	Externalizing behavior
Emotion vocabulary	0.11/−0.05	−0.08/−0.15	0.35***/0.39**	0.11/0.05	−0.05/−0.56***
Language understanding	−0.08	0.00	0.12	0.08	−0.13
Word production	−0.11	0.08	0.19	0.14	−0.09
Sentence production	−0.02	0.17	0.19	0.06	−0.15

Correlations with emotion vocabulary are provided separately for NH and CI, respectively. Correlations with other language indices are provided for CI group only

* *p* < 0.05, ** *p* < 0.01, *** *p* < 0.001 (two tailed)

Finally, we examined the effect of implantation timing on indices of moral emotions and social functioning in the CI group. As can be seen in Table 3, younger age at implantation and longer use of the implant were positively related to pride but not to shame/guilt or any of the indices for social functioning. Although not an initial focus point of this study, we also examined whether implantation timing affected children’s language skills, as previous research indicated [16]. In accordance with previous studies, we found that younger age at implantation and longer time with CI (both corrected for chronological age) were related to better spoken language skills. However, implantation timing was unrelated to emotion language, as indicated by children’s emotion vocabulary (Table 3).

## Discussion

Research shows that moral emotions such as shame, guilt and pride have the ability to promote positive social behavior and to protect against negative social behavior in the NH population [2, 3, 21]. Yet, to our knowledge, this study is the first to examine whether the link between moral emotions and social behavior can already be observed in young children (age 1–5). We examined this in a group of NH children and in a group of children with hearing impairments who had received a CI. The majority of the latter group of children is assumed to have limited opportunities for acquiring social–emotional skills because of restricted communication with their surroundings [36, 37]. Comparing these two groups indirectly provides insight into the influence of socialization on the development of moral emotions.

Our study confirms previous studies [23, 45], which demonstrated that young NH children already display moral emotions, and that this ability increases with age. As expected, children with CI expressed shame/guilt to a lesser extent than their NH peers in response to staged emotion-evoking events (i.e., failing on a mastery task and breaking a toy). In addition, children with CI also showed less pride than NH children when they succeeded on a mastery task. General feedback on children’s performance or behavior was provided by the experimenter, which should have focused children’s attention on their failure or transgression (or in the case of pride, on their success). Nonetheless, children with CI seemed to be less aware than their NH peers of what was expected of them in terms of moral behavior in these situations. On a positive note, as in the NH group, an association was found between age and moral emotions in children with CI. This could imply that moral skills develop along the same lines as in NH children, but at a different pace. Longitudinal studies are required to confirm this.

Expectations concerning the associations between moral emotions and social behavior were largely not met. Firstly, we expected to find a relationship between the extent to which NH children expressed moral emotions and their level of social behavior. Yet, we only found a relationship between moral emotions and positive behavior, not with negative behavior. In other words, a better-developed moral sense did promote positive (i.e., friendly, prosocial) behavior, but did not seem to prevent NH children from displaying negative (i.e., disruptive, externalizing) behavior. It should however be noted that parents of NH children reported quite low levels of behavior problems, which could have masked an association between moral expressiveness and negative behavior. Future studies could try to examine this association in children who show high levels of behavior problems.

Secondly, we expected that the associations between moral and social behavior would be similar in the NH and the CI group. In contrast to this expectation, we found no associations between moral emotions and social behavior in the CI group. As in the NH group, parents of children with CI reported low levels of behavior problems in their children, which could explain the lack of an association between moral emotions and negative behavior. Yet, the absence of a relationship between moral emotions and positive behavior requires some additional attention. Levels of positive as well as negative social functioning were equal in both groups of children, which, in combination with the absence of a relationship between moral emotions and social functioning in children with CI, lead us to question the importance of a delayed development of moral emotions for these children.

The absence of a relationship between moral emotions and social behavior in children with CI could be explained by differences in the ways in which children acquire social–emotional competence. Instilling a moral sense in children is not about teaching them a repertoire of socially appropriate behaviors, rather it is about providing children with the resources to judge their own (intended) behavior as right or wrong. Children are likely to receive explicit feedback on the behavior they display, particularly if this behavior stands out in a positive or a negative way. However, the feedback provided will not always include an evaluation of the morality of children’s behavior. Moreover, children with CI are generally less proficient than NH children in recognizing facial expressions [32, 33] and are also less able to detect differences in intonation in spoken language [34]. In addition, the majority of children with CI have fewer opportunities than their NH peers for incidental learning. They have difficulties overhearing other people’s conversations, particularly in noisy environments [38]. Combined, this could explain why the social skills of children with CI are comparable to those of their NH peers while their moral development is delayed. Moreover, factors related to the rehabilitation program that children in the Netherlands enter after receiving their CI could also play a role. Children with CI receive a lot of attention from adults during their frequent visits to the hospital and in their specialized playgroups. This could provide them with ample models for appropriate social behavior. It is, however, unlikely that these role models also explain how and when to express moral emotions.

An alternative explanation for both the finding of lower levels of moral emotions and of equal levels of social functioning in children with CI as compared to NH children could be group differences in parental attitudes and expectations. Although to our knowledge no studies are currently available on the relation between parenting and social–emotional functioning of children with CI, one previous study did demonstrate that fathers of children with hearing impairments were more protective and less strict in disciplining their children with hearing impairments compared with fathers of NH children [46]. If parents of children with CI indeed have more lenient attitudes toward their children, this could affect their judgment of their children’s behavior. Possibly, these parents rate the level of social competence higher and the level of behavior problems lower compared with how parents of NH children would rate similar behaviors. In addition, we could speculate that a more lenient attitude and lower expectations by parents of children with CI hamper these children’s moral development. Parents who do not correct their children’s inappropriate or rule-breaking behavior will raise children who are unaware of the prevailing moral standards and values. It seems there might be an optimal level of parental power assertion which helps children to internalize moral values. Parents who come on too strong may only foster anger and resentment in their children, leading children to attribute their transgressions to external causes instead of acknowledging their own responsibility and feeling guilty or ashamed. On the other hand, if parents condone bad behavior and do not set firm boundaries, children will also fail to internalize moral values [47]. More research is needed to unravel the relations between parental attitudes, expectations and behavior on the one hand and the development of moral emotions in children with CI on the other hand.

Although the social skills of children with CI seem to develop well, it remains to be seen whether these will continue to develop at the same pace as NH children’s social skills in the absence of equally well-developing emotional skills. As children grow older, more sophisticated social skills are expected, which could draw more heavily on children’s emotional skills. Children with CI are often found to have an impaired ToM [30, 31], which hampers their ability to judge their own behavior from another person’s perspective, and thus could prevent them from experiencing and displaying moral emotions when these are called for. A lack of expressing moral emotions following a transgression does not seem to damage the social relationships of children with CI at this early age. Down the line, peer relations become more important and peers may be less forgiving than parents when children cross the line without showing remorse. There is no doubt that everyone will violate the social norms from time to time. Yet, individuals who display or report feelings of shame or guilt following a transgression are less likely to be socially rejected than those who seem to be indifferent to their wrong doing [2, 48].

To verify whether communication is important for children’s moral and social development we examined one of its components: language. Language did not turn out to be the important determinant of children’s moral emotion expressions we had hypothesized. Children who used more emotion language did not express more moral emotions. Two explanations for this outcome come to mind. First, language does not equal communication. Communication can take on many other forms besides language. Moreover, we only assessed language skills of the children themselves, we did not assess language exchanges between children and important others in their environment. This could have provided much more detailed information on the content of conversations or the way messages are communicated, for example. Second, other factors besides language might be more important for the development of moral emotions. For example, children’s previous experiences regarding consequences of moral transgressions, parental attitudes toward emotions in general or children’s temperament could play a role in the development of moral emotions [9, 47]. Emotion language was, however, associated with more positive social behavior in both groups and with less negative behavior in the CI group. Important to note is that general spoken language skills of children with CI did not influence their emotional or their social functioning. These outcomes support the assumption previously made by other researchers [49] that it is not just the ability to understand and produce general language that is important for social–emotional functioning; it is the ability to understand and use emotion language in daily conversations that is critical for adequate social functioning.

A recent study by Quittner et al. [50] demonstrated that parental behaviors, including language stimulation and maternal sensitivity, had a major impact on language outcomes in children with CI. Nonetheless, parents might continue to experience difficulties conversing about abstract concepts such as emotions with their children with CI, despite these children having adequate concrete language skills. In a study by Zaidman-Zait [51], 39 % of parents reported that they experienced difficulties communicating with their children with CI. A previous study by the current authors demonstrated that children with CI who had well-developed general language understanding still had an impaired theory of mind in comparison to NH age mates [31]. In addition, emotion language was unrelated to implantation timing in the current study, whereas general language was better developed in children who had been implanted at a younger age, or who had been using their implant for a longer time. Together, these findings validate that professionals and parents should actively try to promote the emotion language skills of children with CI to enhance their ability to understand their own and other people’s (emotional) behavior, and to further raise their level of social skill.

The outcomes of this study should be interpreted with caution and represent a preliminary step toward understanding the relations between moral and social behavior. We included a specific subset of children with CI, who were implanted before age three, had hearing parents, and no apparent mental health disorders. Generalization to the whole population of children with CI, of which approximately one-third is reported to have an additional disability [52, 53], is problematic. Furthermore, the correlational and cross-sectional nature of the study precludes drawing any conclusions as to causation. Another point that needs to be addressed concerns the type of data gathered for the purpose of this study. Although parents are a well-informed source regarding their young children’s behavior, their reports on their children’s social and externalizing behavior may have been biased. As discussed above, parents of children with CI may have been overly positive in judging their children’s capacities. Likewise, we cannot be certain that children’s responses to the experimental tasks designed to induce moral emotions were a genuine reflection of how they would behave in a real-life situation. Therefore, adopting a multi-informant approach in future studies is advisable and would resolve some of the limitations present in the current study. Clearly, more (longitudinal) research is necessary to confirm the current findings.

Moreover, the outcomes of this study give rise to new research questions. For example, regarding which factors contribute to children’s moral development. As moral development in children with CI turns out to be impaired it is even more important to gain an understanding of the underlying reasons for this impairment. This might provide us with valuable information on how moral development in these children could be promoted. Attention could be directed to children’s ToM understanding as a likely contributing factor. In addition, it might also be worthwhile to assess parent–child communication more directly instead of by means of children’s language skills (i.e., through observational measures). Communication entails more than just language. Future studies should also incorporate other important aspects of communication, such as nonverbal communication and characteristics of parent–child interactions (e.g., sensitivity, intersubjectivity). Moreover, future studies should address the potentially mediating role of parental attitudes and expectations on children’s moral and social development.

Despite its limitations, this study constitutes an important first step in shedding light on the moral development of young children with CI and with NH, and on its relationship to social functioning. An important finding of this study is that shame and guilt are highly adaptive emotions. They serve the purpose of guiding our social behavior [1, 21], and seem to do so at a very young age already. NH children who displayed higher levels of shame/guilt in response to a transgression were more skilled in interacting with peers and adults.

On the other hand, there is a reason that shame and guilt are often associated with impaired social functioning and are even part of the criteria for several clinical disorders (cf. [54]). Indeed, excessively high levels of shame or guilt may lead to distress and feelings of worthlessness, and hamper the individual’s daily functioning. For instance, proneness to shame and guilt is associated with symptoms of depression and (social) anxiety, and also with aggression [9]. We can conclude that moral emotions are adaptive as long as they are appropriate to the situation and do not become too intense. Therefore, it is important that children learn to find a balance regarding the level of moral emotions they experience in any given situation.

The outcomes of this study support our idea that moral emotions develop in interaction with the social environment. Through their daily social experiences, children learn what acceptable emotion expressions are and with what kind of intensity they should be expressed [55]. In this study, the children with CI represented a group of children with limited opportunities to interact with their social environment. This group indeed turned out to display moral emotions to a lesser extent than a comparison group of NH peers. The notion that moral emotions develop in interaction with the environment is not only important for clinicians who are dealing with children with hearing impairments, but also for clinicians dealing with other clinical groups. For example, children who exhibit conduct problems are also known to have a less well-developed moral sense [5]. Investing in increasing the quality and quantity of social interactions these children have with people in their environment may result in an improved development of moral emotions and consequently, better social functioning.
